# Neuroinvasive West Nile Virus Presenting as Subacute Progressive Quadriparesis and Intractable Pain: A Case Report

**DOI:** 10.1155/crnm/5565739

**Published:** 2026-01-07

**Authors:** Mick B. Reedy, Mohammad Abdul Azeem, Thanujaa Subramaniam, Shahriar Salamat, Howard Rowley, Bradley Beinlich

**Affiliations:** ^1^ Department of Neurology, University of Wisconsin-Madison, Madison, Wisconsin, USA, wisc.edu; ^2^ Department of Pathology, University of Wisconsin-Madison, Madison, Wisconsin, USA, wisc.edu; ^3^ Department of Neurological Surgery, University of Wisconsin-Madison, Madison, Wisconsin, USA, wisc.edu; ^4^ Department of Radiology, University of Wisconsin-Madison, Madison, Wisconsin, USA, wisc.edu

**Keywords:** autopsy, case report, neuroinfectious disease, neuroinvasive West Nile virus, neuromuscular

## Abstract

West Nile virus (WNV) is the most common mosquito‐borne infection in North America; while most cases are asymptomatic, fewer than 1% develop neuroinvasive disease with significant morbidity and mortality. We report a 57‐year‐old man from rural Wisconsin who presented with a 10‐week history of progressive asymmetric quadriparesis and severe intractable pain, preceded by fatigue, shoulder pain, and paresthesias. Neurologic examination demonstrated mild encephalopathy, bulbar involvement, and mixed upper and lower motor neuron signs. MRI showed patchy thoracic cord T2 hyperintensities and diffuse lumbar ventral root enhancement. Electrodiagnostic studies revealed diffuse active denervation and reduced compound muscle action potentials, initially raising concern for amyotrophic lateral sclerosis. Elevated WNV IgM and IgG titers in serum and cerebrospinal fluid confirmed neuroinvasive WNV infection. Despite treatment with corticosteroids and intravenous immunoglobulin, the patient deteriorated and was transitioned to hospice care. Autopsy demonstrated T‐cell–mediated meningoencephalitis with widespread lymphocytic inflammation involving motor neurons, spinal cord, ventral rootlets, and peripheral nerves, consistent with diffuse axonopathy. This case underscores that neuroinvasive WNV may closely mimic motor neuron disease and emphasizes the importance of serologic testing for accurate diagnosis. Management remains supportive, and outcomes can be severe due to extensive central and peripheral nervous system involvement.

## 1. Introduction

West Nile virus (WNV) is the leading cause of domestically acquired arboviral disease in the United States [1]. Recent national surveillance data demonstrate that WNV accounted for 2628 of the 2770 reported arboviral disease cases in 2023, reaffirming its continued predominance among mosquito‐borne pathogens [2]. Long‐term surveillance from 1999 to 2024 confirms the sustained burden and geographic persistence of WNV across the United States [3]. Although transmission through blood products or organ transplantation is rare [4, 5], most infections result from mosquito exposure and demonstrate marked seasonality, with case counts peaking between July and September [6].

Approximately 25% of infected individuals develop symptomatic West Nile fever after an incubation period of 2–14 days and typically present with abrupt, nonspecific symptoms including fever, rash, headache, malaise, myalgias, chills, and emesis [7, 8]. While most cases are mild and self‐limited, fewer than 1% progress to neuroinvasive disease, which may result in severe neurologic complications or death [9]. The neuroinvasive spectrum includes meningitis, encephalitis, or acute flaccid paralysis (AFP), though a broad range of neuromuscular complications has been described [1, 6].

Encephalitis accounts for approximately 50% of neuroinvasive presentations and ranges from mild confusion to coma, often accompanied by tremor and myoclonus [10, 11]. AFP comprises roughly 6% of neuroinvasive cases and typically presents with rapidly progressive asymmetric weakness attributable to anterior horn cell dysfunction, frequently accompanied by bulbar involvement and risk of respiratory failure [10, 12, 13]. Severe limb pain is common and typically precedes weakness, while objective sensory deficits are usually absent [13]. Rare complications include plexopathy, radiculoplexus neuropathy, motor axonopathy, axonal polyneuropathy, and myasthenia‐like syndromes [14].

Here, we present a rare case of neuroinvasive WNV manifesting as subacute progressive quadriparesis with severe generalized pain, closely mimicking motor neuron disease.

## 2. Case Presentation

A 57‐year‐old man from rural Wisconsin presented with a 10‐week history of progressive asymmetric quadriparesis and severe diffuse pain. Symptoms began in late fall with profound fatigue and focal right shoulder pain, followed by worsening diffuse myalgias and bilateral foot paresthesias. Within 1 month, he developed right‐hand weakness that rapidly progressed after a brief 2‐week plateau, culminating in significant weakness of all four extremities by presentation.

One week prior to admission, he was diagnosed at an outside hospital with bilateral pulmonary emboli following evaluation for dyspnea. He also reported unintentional 35‐pound weight loss, severe constipation, and depressive symptoms. He endorsed significant mosquito and tick exposure but denied fever, rash, bladder or bowel incontinence, dysphagia, travel, or recent vaccinations. His past medical history included a motor vehicle accident shortly after symptom onset, resulting in conservatively managed T11–T12 fractures, remote splenectomy, hypertension, and active 20‐pack‐year tobacco use.

On examination, he was tachypneic with slowed mentation and impaired attention but remained oriented. Cranial nerve testing demonstrated mild bilateral abducens palsies and dysarthria. Motor examination revealed marked atrophy with right‐sided fasciculations, spasticity, and proximal‐greater‐than‐distal quadriparesis, most pronounced in the right upper limb. Reflexes were diffusely hyperactive with Hoffman and Babinski signs and sustained right ankle clonus. Sensory examination was intact despite severe reported pain. These findings localized to combined upper and lower motor neuron involvement affecting corticospinal tracts, anterior horn cells, ventral roots, and peripheral motor nerves. Mental status changes raised concern for cerebral or brainstem dysfunction.

### 2.1. Investigations

**Figure 1:**
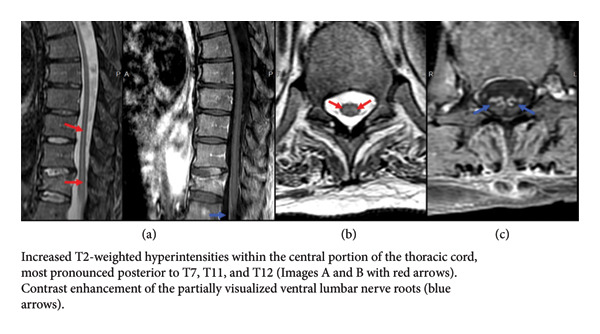
MRI thoracic spine with and without contrast, sagittal (image (a)) and axial views (images (b) and (c), respectively). Increased T2‐weighted hyperintensities within the central portion of the thoracic cord, most pronounced posterior to T7, T11, and T12 (Images (a) and (b) with red arrows). Contrast enhancement of the partially visualized ventral lumbar nerve roots (blue arrows).

Routine laboratory studies were unremarkable. MRI brain and MRA were normal. MRI of the whole spine demonstrated nonenhancing T2 hyperintensities in the distal thoracic cord and diffuse lumbar ventral root enhancement, with stable thoracic fractures (Figure 1). NCS revealed markedly reduced CMAPs with preserved conduction velocities and sensory responses (Table 1), while EMG showed widespread active denervation and chronic reinnervation (Table 2), raising concern for motor neuron disease.

**Table 1:** Nerve conduction studies (NCSs).

Side	Nerve	STIM site	Latency (ms) (normal)	Amplitude (mV) (normal)	Velocity (m/s) (normal)	F‐wave (ms) (normal)
(R)	Sural (s)	Calf	4.5^∗^ (< 4.5)	0.005^∗^ (> 0.006)		NR^∗^ (< 57)

	Peroneal (m)	Ankle	5.7 (< 6.0)	0.6^∗^		
		Fibular head		0.6^∗^	39^∗^	
		Knee		0.6^∗^ (> 2.0)	39^∗^ (> 41)	
	Radial (s)	Forearm	1.8 (< 2.8)	0.029 (> 0.019)		
	Ulnar (m)	Wrist	3.6^∗^ (< 3.6)	1.8^∗^		35.1^∗^ (< 33)
		B elbow		1.6^∗^	54	
		A elbow		1.6^∗^ (> 6.0)	56 (≥ 51)	

*Note:* S, sensory; M, motor; B, below; A, above. 2 Hz repetitive stimulation at rest: No decrement was seen. Lastly, severely reduced right peroneal and ulnar compound muscle action potentials, borderline right sural sensory nerve action potential, and absent F‐wave in right peroneal nerves are observed. The F‐wave is mildly prolonged in the right ulnar nerve. Conduction velocity is minimally prolonged or normal.

^∗^The values are abnormal. The normal range is demonstrated in the parentheses for each abnormal value., e.g., 4.5^∗^ (< 4.5), meaning less than 4.5 is normal and anything else is abnormal.

**Table 2:** Electromyography (EMG).

Side	Muscle	INS	Spontaneous + spikes	Spontaneous + waves/FASC	Rec	MUP duration long	MUP duration short	AMP high	AMP low
(R)	FDI	↑	3+	2+	1+	3+		3+	
(R)	Triceps	↑	3+	3+	2+	3+		3+	
(R)	Deltoid	↑	3+	3+	2+	3+		3+	
(R)	Ant Tib	↑	2+	2+	1+	2+		3+	
(R)	Med Gast	↑	2+	2+	1+	2+		2+	
(R)	Vast Lat	↑	2+	2+	2+	3+		3+	
(L)	FDI	↑	2+	3+	1+	3+		3+	
(L)	Triceps	↑	2+	2+	2+	2+		2+	
(L)	Deltoid	↑	3+	3+	2+	2+		2+	

*Note:* R, right; L, left; Ins, insertional activity; + spikes, positive spikes; + waves/FASC, positive waves/fasciculations; Rec, recruitment; MUP, motor unit potential; AMP, amplitude. Legend: Diffuse active and chronic neurogenic changes with severe motor unit loss were seen in all muscles tested in both upper extremities and the right lower extremity. Left lower extremity testing was not tolerated. Paraspinal muscles were not tested due to the use of therapeutic anticoagulation.

Extensive serum and urine evaluations for autoimmune, infectious, endocrine, nutritional, malignant, and paraneoplastic etiologies were unrevealing. CT pan‐scan was negative for malignancy. CSF showed mild lymphocytic pleocytosis (6 cells/μL), elevated protein (126 mg/dL), ten oligoclonal bands, and an IgG index of 4.40, suggestive of a viral or inflammatory process.

Serum arbovirus testing with plaque‐reduction neutralization revealed elevated WNV IgM and IgG titers, with corresponding elevation in CSF and negative WNV PCR. These findings, together with clinical and imaging features, confirmed neuroinvasive WNV.

### 2.2. Hospital Course

He was empirically treated with intravenous methylprednisolone and IVIg due to initial diagnostic uncertainty. Despite therapy, he deteriorated with progressive encephalopathy, dysphagia requiring gastrostomy, and eventual neuromuscular respiratory failure necessitating tracheostomy. His course was complicated by aspiration pneumonia and *E. coli* gastroenteritis with Shiga toxin, resulting in hypotension.

Pain intensified, spreading from the right shoulder to involve multiple proximal muscle groups, accompanied by diffuse dysesthesias and profound myalgias. Pain was refractory to most therapies, improving only with high‐dose opioids, which worsened respiratory compromise.

By week eight of admission, examination revealed severe hypophonia, asymmetric facial diplegia, jaw myokymia, and progressive limb atrophy with worsening right upper limb weakness and emerging left upper limb involvement. Lower limb strength remained antigravity but proximally weak. Sensory testing remained normal. Due to continued decline, refractory pain, and poor prognosis, he transitioned to hospice after 10 weeks of hospitalization.

### 2.3. Autopsy Findings

**Figure 2:**
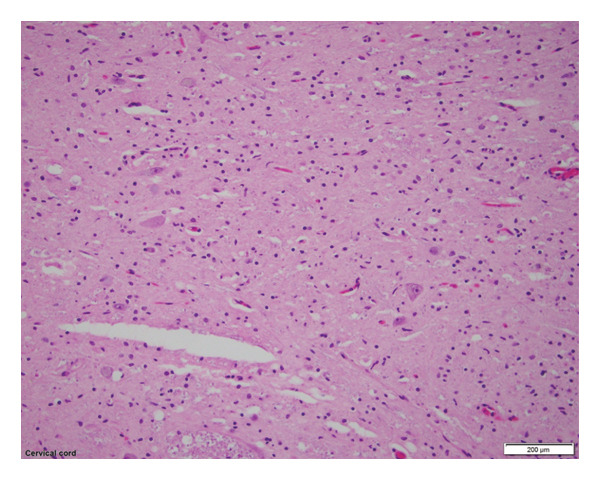
Hematoxylin and eosin stain of cervical spinal ventral gray demonstrating depletion in alpha‐motor neurons and gliosis.

Gross examination showed asymmetric atrophy of the right pyramid and cerebral peduncle with degeneration of the cervical anterior horns. Microscopy revealed diffuse T‐cell–mediated lymphocytic meningoencephalitis with predominant involvement of the brainstem and spinal cord, including severe anterior horn neuron loss (Figure 2). There was no histopathologic evidence of primary motor neuron disease or characteristic inclusions, such as Bunina bodies, skeins, and Lewy body–like inclusions.

**Figure 3:**
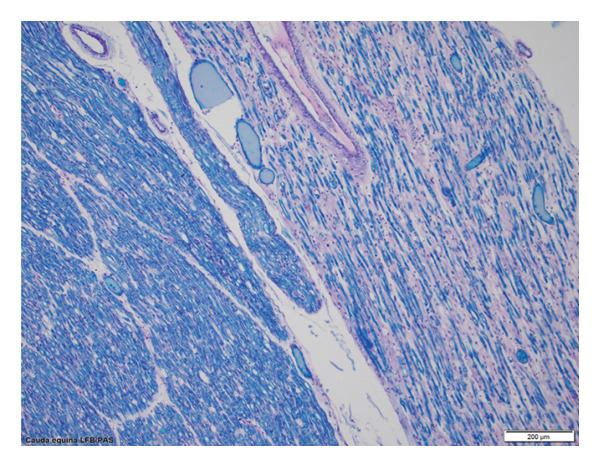
Luxol fast blue stain of cauda equina reveals significant depletion in myelinated fibers of a motor nerve rootlet on the right as compared to a sensory nerve rootlet on the left.

**Figure 4:**
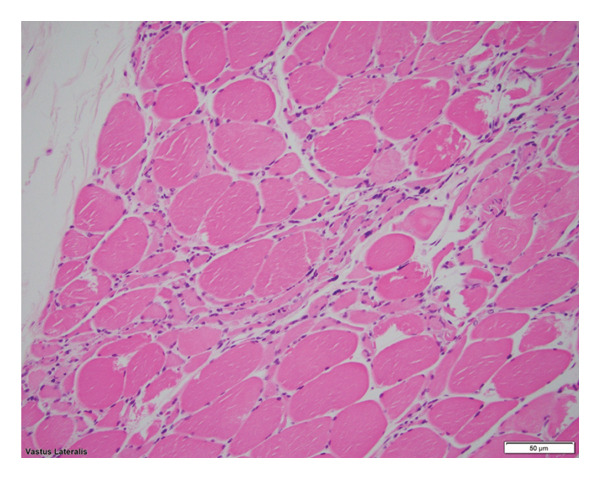
Hematoxylin and eosin stain demonstrating neurogenic atrophy with clusters of highly atrophic fibers and nuclear clusters.

Spinal ventral nerve roots demonstrated lymphocytic and macrophagic infiltration with severe axonal degeneration (Figure 3). Peripheral nerves exhibited mild‐to‐moderate axonopathy, and muscle biopsies showed denervation atrophy (Figure 4), confirming widespread central and peripheral motor pathway involvement consistent with neuroinvasive WNV.

## 3. Discussion

We describe an atypical case of neuroinvasive WNV presenting with progressive quadriparesis and intractable pain, supported by clinical features, neurodiagnostic testing, and laboratory evaluation. The presentation was unusual due to delayed symptom onset, a brief period of stability, and prominent upper motor neuron involvement, which is rarely described in WNV.

Neuroinvasive WNV is classically preceded by a viral prodrome of fever, malaise, headache, and myalgias, with meningeal signs or encephalopathy depending on clinical phenotype [1]. However, prior reports describe cases without prodromal symptoms despite CSF pleocytosis [15]. Our patient similarly lacked classic prodromal findings aside from fatigue, raising the risk of missed or delayed diagnosis in the absence of heightened suspicion.

WNV‐related paralysis is typically asymmetric and attributed to anterior horn cell involvement [16]. Weakness usually develops within days of prodrome but may be delayed, and abrupt deterioration after a period of stability has been documented [17]. Possible mechanisms include persistent viral infection, delayed neuroinvasion, or immune‐mediated injury [17, 18]. Our patient exhibited this pattern, developing rapidly progressive weakness 4 weeks after symptom onset.

Severe limb pain is common and often precedes weakness, though objective sensory deficits are rare [17–19]. Intractable pain significantly affected our patient’s quality of life and limited rehabilitation. Reflex suppression is typical in neuroinvasive WNV with neuromuscular involvement [16, 19, 20]. However, preserved reflexes with combined UMN and LMN signs have been reported [18, 21]. Our patient demonstrated a similar pattern of mixed motor neuron involvement.

Prognosis in AFP varies and is influenced by age, respiratory involvement, and encephalitis [9, 17, 22]. While recovery may occur over months, poor outcomes are associated with low CMAPs and severe deficits [16, 19]. Given our patient’s encephalopathy, respiratory failure, and severely reduced CMAPs, functional recovery was unlikely.

CSF IgM is typically positive within 8 days but may persist for months and does not necessarily reflect acute infection [1, 23]. PCR is frequently negative due to low viremia [24]. Serum IgG titers also poorly define chronicity and may remain elevated for years [25]. While our patient’s titers support recent infection, the precise timing of neuroinvasion cannot be conclusively determined.

CSF findings in neuroinvasive WNV typically include lymphocytic pleocytosis and elevated protein [1]. Minimal lymphocytic pleocytosis has also been documented in other case reports [18, 26]. Evidence suggests there may be an interval reduction in pleocytosis in those who suffer abrupt symptom worsening after clinical stability [17]. MRI findings are often normal but may show ventral root enhancement or cord lesions, as seen in our patient [15, 17, 27]. Electrodiagnostic studies characteristically reveal anterior horn cell dysfunction with reduced CMAPs and preserved conduction velocities, consistent with our findings [12, 17, 18].

Autopsy demonstrated T‐cell–mediated meningoencephalitis with predominant involvement of motor neurons, mirroring previously reported neuroinvasive WNV pathology [7, 12, 13, 21]. The distribution of inflammation and axonopathy explains the patient’s combined UMN and LMN signs, asymmetric weakness, bulbar dysfunction, and encephalopathy. While WNV immunohistochemistry was not performed, such testing has variable sensitivity [7, 21]. Based on the patient’s clinical course, positive serologies, appropriate geographic and seasonal context, and autopsy findings demonstrating inflammatory changes rather than features of motor neuron disease, further testing was deemed unnecessary.

Overall, the autopsy findings support a diffuse immune‐mediated process affecting both central and peripheral motor pathways, consistent with neuroinvasive WNV and correlated with the patient’s clinical trajectory.

## 4. Conclusion

We report a rare case of neuroinvasive WNV presenting with delayed onset, transient stability, rapidly progressive asymmetric quadriparesis, and severe intractable pain refractory to corticosteroids and IVIg. Electrodiagnostic studies demonstrated a motor neuron syndrome, and serum and CSF testing confirmed WNV infection. This case highlights an uncommon presentation of neuroinvasive WNV and emphasizes the importance of considering WNV in atypical motor neuron syndromes, even in the absence of classic viral prodromal symptoms.

## Conflicts of Interest

The authors declare no conflicts of interest.

## Funding

This research received no specific grant from any funding agency in the public, commercial, or not‐for‐profit sectors.

## Data Availability

The deidentified clinical data supporting the findings of this case report are not publicly available due to patient privacy and confidentiality considerations. However, they can be made available upon reasonable request from the corresponding author, contingent upon approval by the appropriate institutional review board.
